# Supplementary material for: Improving models for student retention and graduation using Markov chains
Source: PLoS One. 2023 Jun 26;18(6):e0287775. doi: 10.1371/journal.pone.0287775 (PMC10292706; doi:10.1371/journal.pone.0287775)
Supplement: S1 File — This file contains figures and tables with supplemental analyses to support the main text results. (PDF) [file pone.0287775.s001.pdf]

Supplementary Material

accompanying Improving Models for Student Retention and Graduation using Markov Chains by Tedeschi et al.

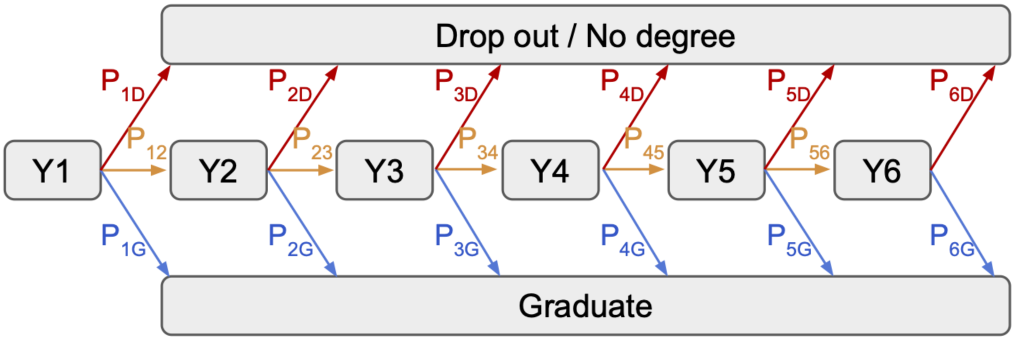

Figure S1. *System diagram to represent students' probabilities of transitioning from one academic state to another. Academic states include matriculated students in their first year (Y1), second year (Y2), and so on through their sixth year (Y6), and departed students who have left the university without a degree (D) or who have left after graduating with a degree (G). The transition probabilities from state i to j are given by* $P_{i,j}$ *and the relevant transitions are depicted with arrows.*

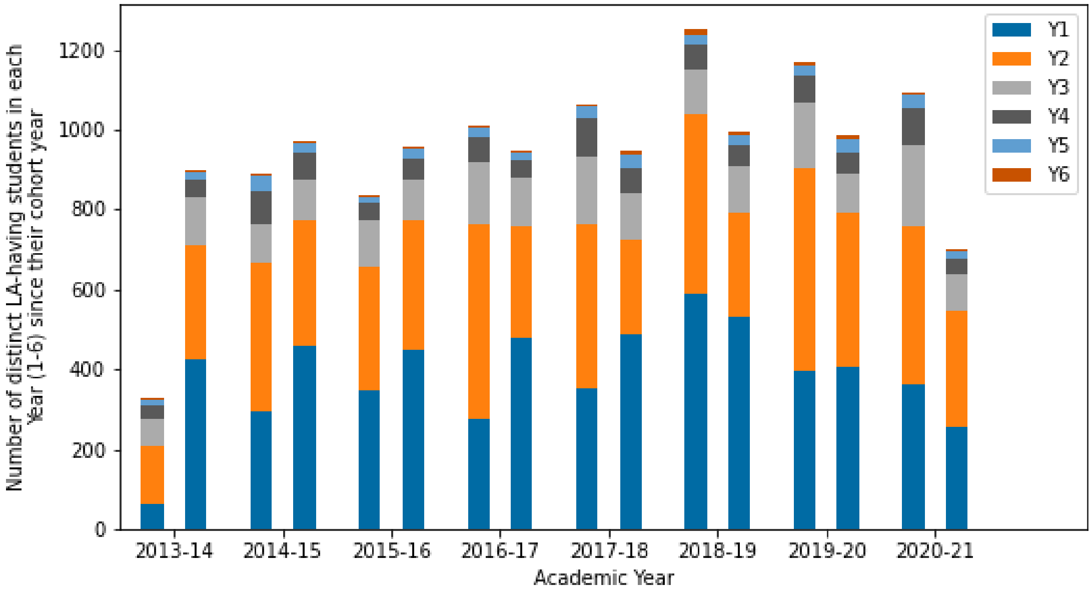

Figure S2. *Learning assistants consistently affect more than 800 students each semester at UNI, with about 20% of these students being in Year 3 or higher. In each pair of stacked bars, the left and right bars represent the fall and spring semesters from that academic year, respectively.*

Table S1. *Numbers of LA-supported and non-LA-supported students and course sections for each high-DFW College of Science course at UNI in the semesters from Fall 2013 to Spring 2021. Entries are numbers of students with numbers of course sections in parentheses.*

		Fa 2013	Sp 2014	Fa 2014	Sp 2015	Fa 2015	Sp 2016	Fa 2016	Sp 2017	Fa 2017	Sp 2018	Fa 2018	Sp 2019	Fa 2019	Sp 2020	Fa 2020	Sp 2021	Total
**College Physics I (PHYS 111)**	**LA**	0 (0)	0 (0)	0 (0)	0 (0)	0 (0)	0 (0)	79 (1)	123 (1)	0 (0)	0 (0)	0 (0)	0 (0)	0 (0)	0 (0)	0 (0)	0 (0)	202 (2)
	**no-LA**	395 (6)	436 (4)	308 (6)	416 (5)	265 (5)	411 (5)	164 (3)	218 (3)	267 (4)	355 (4)	256 (5)	387 (4)	277 (4)	385 (4)	249 (3)	403 (4)	5192 (69)
**College Physics II (PHYS 112)**	**LA**	0 (0)	122 (1)	0 (0)	0 (0)	0 (0)	0 (0)	114 (1)	102 (1)	0 (0)	0 (0)	0 (0)	99 (1)	0 (0)	0 (0)	64 (1)	0 (0)	501 (5)
	**no-LA**	137 (2)	137 (2)	175 (2)	167 (2)	151 (2)	165 (2)	30 (1)	42 (1)	132 (2)	146 (2)	126 (2)	61 (1)	121 (2)	138 (2)	83 (1)	146 (1)	1957 (27)
**University Physics I (PHYS 211)**	**LA**	84 (2)	166 (4)	42 (1)	42 (1)	42 (1)	123 (3)	123 (3)	207 (5)	210 (5)	210 (5)	149 (4)	123 (3)	166 (4)	83 (2)	129 (4)	144 (3)	2043 (50)
	**no-LA**	213 (6)	182 (5)	134 (4)	304 (8)	175 (5)	219 (6)	93 (4)	189 (7)	28 (2)	214 (7)	50 (2)	264 (7)	75 (2)	259 (7)	70 (2)	250 (8)	2719 (82)
**University Physics IA (PHYS 211A)**	**LA**	0 (0)	0 (0)	0 (0)	0 (0)	0 (0)	0 (0)	42 (1)	0 (0)	0 (0)	0 (0)	16 (1)	64 (2)	17 (1)	0 (0)	35 (1)	82 (2)	256 (8)
	**no-LA**	114 (3)	179 (5)	111 (3)	189 (5)	116 (4)	258 (7)	102 (3)	151 (5)	112 (3)	110 (4)	22 (1)	42 (1)	35 (1)	88 (3)	0 (0)	0 (0)	1629 (48)
**University Physics II (PHYS 212)**	**LA**	65 (2)	81 (2)	82 (2)	121 (3)	69 (2)	167 (4)	90 (2)	80 (2)	25 (1)	76 (2)	115 (3)	129 (4)	81 (2)	190 (5)	90 (3)	83 (2)	1544 (41)
	**no-LA**	261 (8)	326 (10)	333 (9)	153 (5)	301 (9)	171 (6)	418 (12)	312 (10)	420 (13)	260 (8)	294 (9)	144 (4)	279 (8)	118 (4)	261 (7)	246 (7)	4297 (129)
**Precalculus (MATH 111)**	**LA**	0 (0)	0 (0)	36 (1)	0 (0)	78 (2)	0 (0)	0 (0)	0 (0)	0 (0)	0 (0)	0 (0)	0 (0)	0 (0)	0 (0)	0 (0)	0 (0)	114 (3)
	**no-LA**	440 (13)	216 (6)	425 (12)	131 (4)	426 (12)	174 (5)	238 (8)	102 (4)	218 (7)	100 (4)	211 (6)	85 (3)	409 (14)	97 (3)	519 (14)	103 (3)	3894 (118)
**Calculus A (MATH 171)**	**LA**	0 (0)	0 (0)	0 (0)	0 (0)	0 (0)	0 (0)	0 (0)	0 (0)	0 (0)	0 (0)	268 (7)	0 (0)	0 (0)	0 (0)	0 (0)	0 (0)	268 (7)
	**no-LA**	348 (9)	279 (7)	372 (9)	301 (8)	282 (8)	275 (8)	244 (7)	143 (4)	248 (7)	132 (4)	0 (0)	151 (4)	333 (9)	197 (6)	332 (9)	248 (6)	3885 (105)
**Calculus II (MATH 182A)**	**LA**	0 (0)	0 (0)	0 (0)	0 (0)	0 (0)	0 (0)	0 (0)	0 (0)	0 (0)	0 (0)	0 (0)	0 (0)	0 (0)	29 (1)	0 (0)	0 (0)	29 (1)
	**no-LA**	0 (0)	165 (5)	0 (0)	202 (6)	35 (1)	369 (11)	70 (2)	269 (8)	66 (2)	314 (8)	77 (2)	303 (9)	77 (2)	228 (6)	103 (3)	289 (7)	2567 (72)
**Elements of Multivariable Calculus and Differential Equations (MATH 211)**	**LA**	0 (0)	0 (0)	44 (1)	110 (3)	144 (4)	132 (3)	89 (3)	119 (3)	138 (4)	110 (3)	146 (4)	39 (1)	0 (0)	102 (3)	0 (0)	0 (0)	1173 (32)
	**no-LA**	137 (4)	77 (4)	85 (3)	0 (0)	0 (0)	0 (0)	34 (1)	0 (0)	0 (0)	0 (0)	0 (0)	70 (2)	156 (4)	0 (0)	154 (4)	117 (3)	830 (25)

Table S2. *Total numbers of students in high-attrition lower-division UNI College of Science courses who contribute to the estimates of the six-year graduation rate from the Markov model. Top row gives total numbers of students with College of Science majors overall (All), AALANA students, and first-generation students. Bottom row gives the numbers of students with College of Science majors who have had a learning assistant-assisted (LA-assisted) course at some point during their time at UNI.*

	**All**	**AALANA**	**First Generation**
Total	1395	228	268
LA	457	62	86

Table S3. *Estimates of confidence intervals for six-year graduation rates (%) are not sensitive to increasing the size of the bootstrap ensembles relative to the main text (1,000 replicates). A traditional calculation for the Fall 2013 cohort is used here as an example.*

**Ensemble size**	**2.5th percentile**	**Median**	**97.5th percentile**
1000	66.4	70.6	74.8
2000	66.2	70.5	75.0
4000	66.2	70.5	75.0
8000	66.2	70.6	75.2

Table S4. *Learning assistant (LA) support is associated with increases in year-to-year persistence (%) for science majors. Persistence rates are estimated as the Markov transition probability using all available data (Fall 2013 - Summer 2021). Numbers are rounded to the nearest percentage point.*

**Transition**	**No-LA**	**LA**	**Difference (LA - no-LA)**
Y1→Y2	87	95	+9
Y2→Y3	91	93	+2
Y3→Y4	91	93	+3

Table S5. *Learning assistant (LA) support is associated with increases in year-to-year persistence (%) for AALANA science majors. Persistence rates are estimated as the Markov transition probability using all available data (Fall 2013 - Summer 2021). Numbers are rounded to the nearest percentage point.*

**Transition**	**No-LA**	**LA**	**Difference (LA - no-LA)**
Y1→Y2	84	92	+9
Y2→Y3	88	92	+4
Y3→Y4	89	94	+5

Table S6. *Learning assistant (LA) support is associated with increases in year-to-year persistence (%) for first-generation science majors. Persistence rates are estimated as the Markov transition probability using all available data (Fall 2013 - Summer 2021). Numbers are rounded to the nearest percentage point.*

**Transition**	**No-LA**	**LA**	**Difference (LA - no-LA)**
Y1→Y2	86	96	+9
Y2→Y3	90	93	+3
Y3→Y4	90	91	+1
